# The Management of Extrahepatic Portal Vein Aneurysms: Observe or Treat?

**DOI:** 10.1155/1996/37169

**Published:** 1996

**Authors:** Philip D. Feliciano, Joseph J. Cullen, John D. Corson

**Affiliations:** Department of Surgery University of Iowa College of Medicine Iowa City Iowa 52242 USA

## Abstract

A case of a 70 year old man who was found to have an extrahepatic portal vein aneurysm during an evaluation for hematuria is reported. Extrahepatic portal vein aneurysms are rare with only twenty cases reported in the literature. Typically, patients present with hemorrhage requiring surgical exploration or the aneurysm is discovered during evaluation of another abdominal process. Management includes careful follow-up in the asymptomatic patient without underlying liver disease or portal hypertension.


*HPB Surgery*, 1996, Vol.10, pp. 113–116
Reprints available directly from the publisher
Photocopying permitted by license only

© 1996 OPA (Overseas Publishers Association)
Amsterdam B.V. Published in The Netherlands
by Harwood Academic Publishers
Printed in Malaysia

### CASE REPORT
### The Management of Extrahepatic Portal Vein Aneurysms: Observe or Treat?

PHILIP D. FELICIANO, JOSEPH J. CULLEN and JOHN D. CORSON

Department of Surgery, University of Iowa College of Medicine, Iowa City, Iowa, 52242.

*(Received 30 August 1995)*

A case of a 70 year old man who was found to have an extrahepatic portal vein aneurysm during an evaluation for hematuria is reported. Extrahepatic portal vein aneurysms are rare with only twenty cases reported in the literature. Typically, patients present with hemorrhage requiring surgical exploration or the aneurysm is discovered during evaluation of another abdominal process. Management includes careful follow-up in the asymptomatic patient without underlying liver disease or portal hypertension.

KEY WORDS: Portal vein venous aneurysm portal hypertension

The first report of an extrahepatic portal vein aneurysm in 1956 described a twenty-one year old female with cirrhosis who presented with rupture of the aneurysm into the common bile duct[1]. In contrast to arterial aneurysms, primary venous aneurysms are uncommon. Venous aneurysms have been described in the popliteal, jugular and saphenous veins, but occur rarely in the superior vena cava, axillary veins, intracranial veins, or portal vein. Prior to 1980, the majority of portal vein aneurysms were identified during laparotomy, however with the liberal use of abdominal ultrasound and the advent of computerized axial tomography (CT), these aneurysms may be identified more frequently with these diagnostic techniques. We report the incidental finding of a large extrahepatic portal vein aneurysm discovered inadvertently during an evaluation for hematuria. The literature regarding the management and treatment of portal vein aneurysms is reviewed.

### CASE REPORT

A 70-year old white male presented with a chief complaint of hematuria. Additionally, the patient reported a recent weight loss of 30 lbs. over the past six months, but denied any symptoms of abdominal pain, nausea, vomiting, fever, chills or change in bowel function, or history of alcohol abuse. On physical examination the patient appeared well nourished and was normotensive without jaundice, or stigmata of cirrhosis. His abdomen was soft and nontender, without hepatosplenomegaly or palpable abdominal masses. Rectal exam revealed no masses and there was no blood in the stool. Laboratory data revealed the following (S.I. values): hemoglobin 141g/L; white blood cell count $8.3 \times 10^9$/L; total bilirubin 141 μmoles/L; blood urea nitrogen 0.55 millimoles/L; creatinine 97 μmoles/L; alkaline phosphatase $0.9 \times 10^6$ katal/L; glucose 4.1 millimoles/L.

A work-up was initiated with an intravenous pyelogram which revealed indistinct filling of the upper pole of the right kidney. An abdominal ultrasonograph identified a cystic mass between the gallbladder and the head of the pancreas arising posterior to the duodenum. The lesion was anechoic and measured $5.7 \times 6.0$ cm. A color duplex scan confirmed the presence of low blood flow within the lesion. A CT scan confirmed the presence of a mass in the right upper quadrant near the second portion of the duodenum, anterior to the inferior vena cava, right kidney and right adrenal gland (Fig.1).

Esophagogastroduodenoscopy revealed no evidence of foregut pathology or varices. Arteriography

*Correspondence to*: Joseph J. Cullen, M.D., Department of Surgery, 4622 JCP, University of Iowa Hospitals and Clinics, Iowa City, Iowa, 52242.

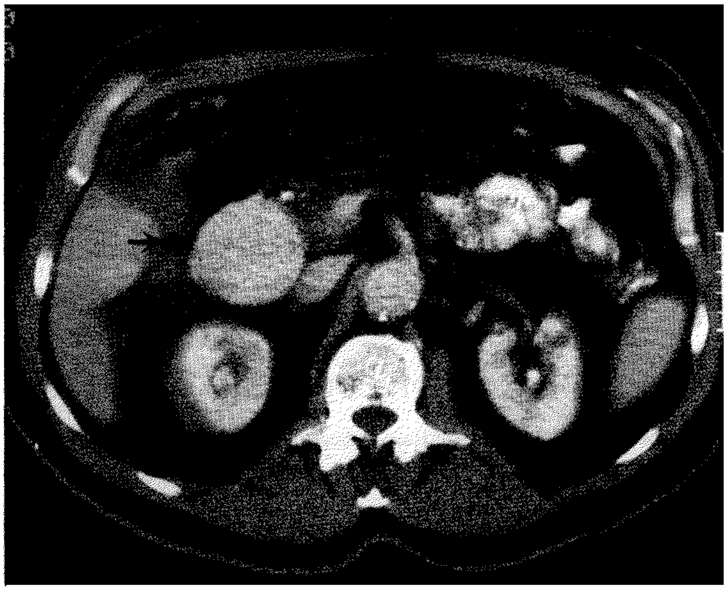

**Figure 1** Contrast enhanced abdominal CT, showing a mass in the right upper quadrant near the second portion of the duodenum, anterior to the inferior vena cava and right kidney.

verified the presence of an extra-hepatic portal vein aneurysm seen in the late venous phase of the study (Fig. 2). Despite the large size of the aneurysm, we elected to observe the aneurysm due to the age of the patient and absence of symptoms.

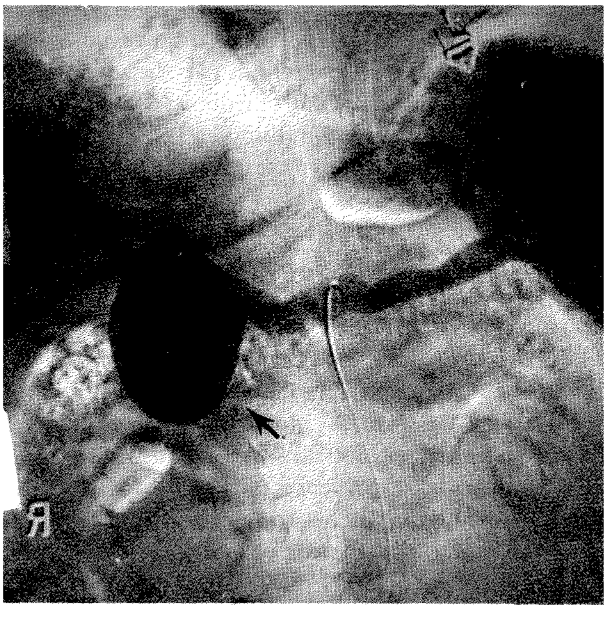

**Figure 2** Late venous phase of the mesenteric arteriography demonstrating the presence of an extrahepatic portal vein aneurysm.

### DISCUSSION

Extrahepatic portal vein aneurysms are rare with 20 cases having been reported in the literature. The age varies from 5 to 70 years with portal hypertension and cirrhosis occuring in 40% and 15%, respectively. Reported cases of extrahepatic portal vein aneurysms are listed in Table 1.

The etiology of portal vein aneurysms in unclear. Some authors have suggested that portal hypertension causes portal vein aneurysms secondary to the increased intraluminal pressure on the relatively thin portal vein wall[2,3]. Endophlebohypertrophy and endophlebosclerosis may also play a role in the pathogenesis of venous aneurysms. Histologic examination of a venous aneurysm reveals a decrease in the number and size of muscle and elastic fibers within the vessel wall, and fragmentation of the internal elastic lamella with replacement by fibrous connective tissue[4]. Other conditions which have been proposed as possible etiologies of a portal vein aneurysm include trauma and pancreatitis[5]. A congenital etiology for portal vein aneurysms has also been suggested by number of authors[6–9]. Although variations in the embryonic development of the portal vein have been described, including double or hypoplastic portal vein formation, aneurysmal formation of the extrahepatic portal vein appears to be extremely unusual in children. However, a portal vein aneurysm has been iden-

**Table 1** Reported cases of extrahepatic portal vein aneurysm

| *Author* | *Year* | *Presentation* | *Portal Hyper-tension* | *Cirrhosis* | *Size (cm)* | *Treatment* |
|---|---|---|---|---|---|---|
| Barzilai[1] | 1956 | UGI bleed | Yes | Yes | 2 | Cholecystostomy |
| Leonsins[2] | 1960 | UGI bleed,pain | Yes | No | 8×4 | Splenectomy |
| Sedgwick[11] | 1960 | UGI bleed | Yes | Yes | 5 | Splenorenal shunt and cholecystojejunostomy |
| Hermann[6] | 1965 | UGI bleed | Yes | No | 4×6 | Portocaval shunt |
| Liebowitz[12] | 1967 | Pain | No | No | 8 | Splenectomy |
| Thomas[7] | 1967 | UGI bleed, pain | Yes | Yes | 8×6 | Expired due to aneurysm rupture |
| | 1967 | UGI bleed | Yes | No | 3 | Portocaval shunt |
| Vine[13] | 1979 | Fever | ? | ? | 3 | Observation |
| Ishikawa[14] | 1980 | ? | Yes | ? | ? | ? |
| Kane[15] | 1982 | ? | Yes | Yes | ? | ? |
| Schild[5] | 1982 | Incidental | No | No | 4 | Observation |
| Fahrendorf[16] | 1986 | Incidental | No | No | 4 | Observation |
| Boyez[17] | 1986 | Pain | No | No | 4 | Observation |
| Thompson[8] | 1986 | Pain | No | No | 6 | Cholecystectomy |
| Andoh[9] | 1988 | pain | No | No | 6×8 | Splenectomy, partial resection of aneurysm |
| Lee[18] | 1989 | Pain | No | No | 1.9 | Observation |
| Baker[19] | 1990 | Pain | No | No | 8 | Splenectomy, partial resection |
| Hagiwara[20] | 1991 | Pain | No | No | 2.7 | Observation |
| Dognini[21] | 1991 | Pain | No | No | 2.4 | ERCP |
| Glazer[22] | 1991 | Pain | Yes | No | 7 | Thrombectomy; aneurysmorrhaphy |
| Present case | 1994 | Incidental | No | No | 5 | Observation |

?, unknown or not described in report; UGI, upper gastrointestinal; ERCP, endoscopic retrograde cholangiopancreatography.

tified by routine obstetric sonography in a 37 week fetus which supports a congenital etiology for some portal vein aneurysms[10].

Patients reported with portal vein aneurysm prior to 1980 typically presented with upper gastrointestinal bleeding requiring urgent surgical exploration. Currently, patients with portal vein aneurysms are diagnosed during workup of another abdominal process. Useful diagnostic modalities include ultrasonography, color-doppler flow imaging, venous phase mesenteric angiography, magnetic resonance imaging, and radionuclide hepatic scintigraphy. A duplex scan is a useful tool since it combines anatomical delineation of surrounding structures with an assessment of the biphasic wave flow velocity commonly observed in the portal venous system. An abdominal CT provides better anatomical outline of the aneurysm which typically appears as a well-circumscribed oval mass. Venous phase angiography may provide additional information, however intraluminal thrombus can obscure the aneurysm, making the diagnosis more difficult.

Treatment of a portal vein aneurysms depends on the initial clinical presentation of the patient. Patients presenting with upper gastrointestinal hemorrhage have required surgical intervention. Hagiwara *et al.* have suggested that careful follow-up is sufficient in patients with asymptomatic portal vein aneurysms provided there is no underlying liver disease or portal hypertension.

In summary, portal vein aneurysms are extremely rare. Patients are likely to present with upper gastrointestinal bleeding and pain, however portal vein aneurysms can be discovered during evaluation of other abdominal processes. Duplex scan, abdominal CT, and venous phase mesenteric angiography are important diagnostic modalities in the evaluation of these aneurysms. Treatment should be based primarily on the clinical presentation of the patient. If patients with portal vein aneurysms are asymptomatic, observation is a reasonable alternative. Surgical alternatives to be considered in the treatment of portal vein aneurysms include portal decompression, splenectomy, thrombectomy, and aneurysmorraphy.

### REFERENCES

1. Barzilai R, Kleckner M. S. (1950) Hemocholecyst following ruptured aneurysm of portal vein. *Arch Surg* **72**: 725–727.
2. Leonsins A. J, Siew S. (1960) Fusiform aneurysmal dilatation of the portal vein. *Postgrad Med J* **36**: 570–574.
3. Ohnishi K, Nakayama T, Saito M, *et al.* (1984) Aneurysm of the intrahepatic branch of the portal vein. Report of two cases. *Gastroenterology* **86**: 169–173.
4. Friedman S. G, Krishnasatry K. V, Doscher W, *et al.* (1990) Primary venous aneurysms. *Surgery* **108**: 92–95.

5. Schild H, Schweden F, Braun B. (1982) Aneurysm of the superior mesenteric vein. *Radiology* **145**: 641–642.
6. Hermann RE, Shafter W. H. (1965) Aneurysm of the portal vein and portal hypertension: First reported case. *Ann Surg* **162**: 1101–1104.
7. Thomas T. V. (1967) Aneurysm of the portal vein. Report of two cases, one resulting in thrombosis and spontaneous rupture. Surgery **61**: 550–555.
8. Thompson P. B, Oldham K. T, Bedi D. G *et al.* (1986) Aneurysmal malformation of the extrahepatic portal vein. *Am J Gastroenterol* **81**: 695–697.
9. Andoh K, Tanohata K, Asakura K, *et al.* (1988) CT demonstration of portal vein aneurysm. *J. Comput Assist Tomogr* **12**: 325–327.
10. Gallagher D. M, Leiman S, Hux C. H. (1993) In utero diagnosis of a portal vein aneurysm. *J Clin Ultrasound* **21**: 147–151.
11. Sedgwick C. E. (1960) Cisternal dilatation of portal vein associated with portal hypertension and partial biliary obstruction. *Lahey Clin Bull* **11**: 234–237.
12. Leibowitz HR, Rousselot L. M. (1967) Saccular aneurysm of portal vein with agnongenic myeloid metaplasia. NY State *J Med* **67**: 1443–1447.
13. Vine HS, Sequieira J. C, Widrich WC, *et al.* (1979) Portal vein aneurysm. *Am J Roentgenol* **132**: 557–560.
14. Ishikawa T, Tsukune Y, *et al.* (1980) Venous abnormalities in portal hypertension demonstrated by CT. AJR **134**: 271–276.
15. Kane R. A, Katz S. G. (1982) The spectrum of sonographic findings in portal hypertension: A subject review and new observations. *Radiology* ;**142**: 453–458.
16. Fahrendorf V. G, Fishedick A. R. (1986) Aneurysma der vena portae. *ROFO* **144**: 732–734.
17. Boyez M, Fourcade Y, *et al.* (1986) Aneurysmal dilatation of the portal vein: a case diagnosed by real-time ultrasonography. *Gastrointest Radiol* **11**: 319–321.
18. Lee H. C, Yang Y. C, *et al.* (1980) Aneurysmal dilatation of the portal vein. *J Pediatr Gastroenterol Nutr* **8**: 387–389.
19. Baker B, Nepute J. (1990) Computed tomography demonstrated of acute thrombosis of a portal vein aneurysm. *Missouri Med* **100**: 228–230.
20. Hagiwara H, Kasahara A, *et al.* (1991) Extrahepatic portal vein aneurysm associated with a tortuous portal vein. *Gastroenterology* **100**: 818–821.
21. Dognini L, Carreri A. L, Moscatelli G. (199) Aneurysm of the portal vein: ultrasound and computed tomography identification. *J Clin Ultrasound* 1991;**19**: 178–182.
22. Glazer S, Gaspar MR, Esposito V, *et al.* (1992) Extrahepatic portal vein aneurysm: Report of a case treated by thrombectomy and aneurymorrhaphy. *Ann Vasc Surg* **6**: 338–342.

